# Association of Area Deprivation Index with perinatal and COVID-19 outcomes during the early pandemic: a secondary analysis of a prospective cohort study

**DOI:** 10.1080/14767058.2025.2548416

**Published:** 2025-08-25

**Authors:** Abigail Bell, Megan Foeller, Candice Woolfolk, Emily Diveley, Fan Zhang, Amy Scheffer, Ruizhi Huang, Daniel Jackson, Nandini Raghuraman, Ebony Carter, Indira U. Mysorekar, Jeannie C. Kelly

**Affiliations:** a Department of Obstetrics and Gynecology, Washington University School of Medicine, St. Louis, MO, USA; b Department of Obstetrics and Gynaecology, St Alphonsus Regional Medical Center, Boise, ID, USA; c Division of OB/GYN Clinical Research, Department of Obstetrics and Gynecology, Washington University School of Medicine, St. Louis, MO, USA; d Division of Maternal-Fetal Medicine and Ultrasound, Department of Obstetrics and Gynecology, Washington University School of Medicine, St. Louis, MO, USA; e Mercy Clinic Maternal and Fetal Medicine, St. Louis, MO, USA; f Department of Medicine, Section of Infectious Diseases, Baylor College of Medicine, Houston, TX, USA; g Department of Molecular Virology and Microbiology, Baylor College of Medicine, Houston, TX, USA

**Keywords:** Neighborhood deprivation, COVID-19, preterm birth, social determinants of health, Area Deprivation Index

## Abstract

**Objective:**

We sought to evaluate the relationship between neighborhood-level deprivation and perinatal outcomes, particularly preterm birth, as well as COVID-19 outcomes among pregnant patients during the early pandemic.

**Methods:**

We performed a secondary analysis of a prospective longitudinal cohort study that investigated the impact of prenatal COVID-19 exposure on perinatal outcomes. Pregnant patients were recruited 12/23/20–7/18/22 and serially assessed for COVID-19 exposure during pregnancy, with serum antibody testing, electronic medical record review, and patient report. For this secondary analysis, address at the time of delivery was used to categorize patients into low ADI (≤50^th^ percentile Area Deprivation Index, indicating low level of deprivation) and highest ADI (≥75^th^ percentile, indicating highest level of deprivation) groups. The primary outcome was preterm birth, at <37 weeks, <34 weeks, and <28 weeks; secondary outcomes included other perinatal complications as well as COVID-19 outcomes. To further evaluate the effect of SARS-CoV-2 infection on pregnancy outcomes, we completed a subgroup analysis evaluating perinatal outcomes among patients who were SARS-CoV-2-positive at any point in pregnancy. Finally, we stratified patients by race to determine whether racism further impacted outcomes of SARS-CoV-2 infection, preterm birth, and gestational age at delivery.

**Results:**

306 patients were included: 124 in the low ADI group and 182 in the highest ADI group. Baseline characteristics differed between groups, including notably lower rates of COVID-19 vaccination in the highest ADI group (24.2% vs 54.0%, *p* < 0.01). Overall, preterm birth rates did not differ between highest and low ADI groups (23.1% vs 17.7%, respectively, *p* = 0.26); however, highest ADI was significantly associated with higher rates of preterm birth before 28 weeks (12.1% vs 4.0%, *p* = 0.01) and before 34 weeks (12.6% vs 5.7%, *p* = 0.04) even after adjusting for obesity and tobacco use (<28 weeks aOR 0.26, 95% CI 0.07–0.73; <34 weeks aOR 0.35 95% CI 95% 0.14–0.87). The highest ADI group was more likely to develop SARS-CoV-2 infection (59.3% vs 41.9% *p* < 0.01), and more likely to be hospitalized due to COVID-19 (3.9% vs 0.0%, *p* = 0.04). In a subgroup analysis of patients who were SARS-CoV-2-positive during pregnancy, there were no differences found between ADI groups. After stratification by race, there were no statistically significant differences in SARS-CoV-2 infection rates, average gestational age at delivery, or preterm delivery rates between highest and low ADI groups for any race.

**Conclusion:**

Patients in the highest ADI group, therefore living in the most deprived neighborhoods, were more likely to experience extremely preterm birth, contract SARS-CoV-2 infection, and become hospitalized due to COVID-19. The interplay of social determinants of health and perinatal outcomes must be better studied to target interventions to modifiable risk factors.

## Introduction

The novel Coronavirus Disease 19 (COVID-19) pandemic disproportionately affected patients living in the most disadvantaged neighborhoods in the United States, leading to higher rates of SARS-CoV-2 infection as well as increased morbidity and mortality from COVID-19 [1–3]. The complex interplay of many social and structural drivers of health likely underlies this association, including factors of race, ethnicity, socioeconomic status, healthcare access, occupational exposure, and neighborhood characteristics such as overcrowding. Among pregnant patients, neighborhood deprivation has also been associated with increased rates of perinatal complications, including preterm birth [4,5], low birthweight (<2500 g) at term [5], postpartum hypertension [6], post-induction cesarean delivery [7], and impaired glucose tolerance in early pregnancy [8]. However, the relationship of neighborhood deprivation during the COVID-19 pandemic with clinical outcomes among pregnant patients has not fully been investigated [9].

The Area Deprivation Index (ADI) is a measure that accounts for factors of income, education, employment, and housing to provide a surrogate for neighborhood-level social and structural drivers of health in the United States [10,11]. ADI provides a national percentile based on U.S. Census Bureau blocks and has been used in obstetrics and COVID-19 research [1,6,7,12–16] but has rarely been applied to perinatal outcomes during the COVID-19 pandemic [9].

We hypothesize that the COVID-19 pandemic worsened inequities in obstetric care by amplifying barriers faced by disadvantaged communities. To inform future efforts to reduce these disparities, we analyzed a prospective cohort of obstetric patients during the pandemic, examining perinatal and COVID-19 outcomes in relation to neighborhood deprivation (ADI).

## Methods

We performed a secondary analysis of a prospective longitudinal cohort study that investigated the impact of COVID-19 exposure in pregnancy on perinatal outcomes [17]. Patients with a confirmed intrauterine singleton pregnancy between December 23, 2020 and July 18, 2022 were enrolled at two urban tertiary care centers (one academic and one community practice). In the primary analysis, patients were divided into exposed and unexposed cohorts based on SARS-CoV-2 serum IgM/IgG antibody testing (in unvaccinated patients) at enrollment and at each trimester, as well as electronic medical record chart review, patient report, and universal antigen testing at delivery. Medical and sociodemographic histories, including COVID-19 vaccination status and details of SARS-CoV-2 infection, as applicable, were collected at enrollment and updated throughout pregnancy. Neonatal and maternal outcomes were recorded at delivery.

For this secondary analysis, the address of residence recorded in the electronic medical record within one month of the end of pregnancy was used to categorize patients by ADI. ADI is a measure of neighborhood deprivation incorporating domains of income, education, employment, and housing quality into national percentile ranks [10,11]. Higher rank indicates a higher level of neighborhood deprivation. National percentiles were used rather than state-only percentiles to include patients who travel from a nearby state and to improve generalizability. Patients with no recorded address within one month of delivery were excluded. Exclusions also included those living in areas of ADI suppression, which are defined as Census block groups containing any of the following: less than 100 people, less than 30 housing units, more than 33% of the population living in group quarters, or Census data labeled as N/A or missing in the core component variables [10]. This study was approved by our program’s Institutional Review Board (reference number 202012075–1001; approved April 13, 2023).

Perinatal outcomes were compared between patients with ADI percentiles ≤ 50% (“low” ADI, indicating lower levels of deprivation) and ≥ 75% (“highest” ADI, indicating highest levels of deprivation); patients with ADI percentiles between 50% and 75% were excluded. These cutoffs were selected with the intention of comparing the most deprived quartile to those less deprived. All patients, regardless of SARS-CoV-2 seropositivity, were included, with a subsequent SARS-CoV-2-positive subgroup analysis described below. Descriptive summaries of baseline demographics were calculated using χ^2^ or Fisher’s exact test for categorical variables and Student’s *t*-test or Mann–Whitney U test for continuous variables, as appropriate. Data analyses were generated using SAS version 9.4 [18,19].

The *a priori* primary outcome was preterm birth, either spontaneous or iatrogenic, defined as delivery at <37, <34, and <28 weeks. Secondary outcomes included mode of delivery; maternal complications during delivery, including hemorrhage, chorioamnionitis or endomyometritis, shoulder dystocia, blood transfusion, and maternal death; APGAR scores at one and five minutes of life; low and very low neonate birth weight, defined as <2500 and <1500 grams, respectively; and neonatal complications following delivery, including necrotizing enterocolitis, chromosomal abnormality, intracranial hemorrhage, NICU admission, and neonatal death. Control variables that did not improve model fit were excluded, including COVID-19 infection, hospitalization, and hospital site.

COVID-19 morbidity was assessed between the highest and low ADI groups with outcomes of SARS-CoV-2 infection, hospitalization due to COVID-19, and number of days hospitalized. The role of ADI in COVID-19 morbidity was further assessed by conducting a subgroup analysis of the same outcomes as above among those with confirmed SARS-CoV-2 exposure (SARS-CoV-2 seropositivity) between the highest and low ADI groups.

For outcomes of SARS-CoV-2 infection, preterm birth, and gestational age at delivery, we further stratified patients by race. As race is a social construct without biologic basis, we sought to determine whether racism further impacted outcomes for patients in different ADI groups.

## Results

448 patients with a confirmed intrauterine singleton pregnancy between December 23, 2020 and July 18, 2022 were enrolled. 159 patients in the unexposed cohort and 231 patients in the exposed cohort (of which 54 patients crossed over from the unexposed cohort to the exposed cohort during pregnancy following enrollment) were retained through delivery for a total of 390 patients.

**Figure 1.**
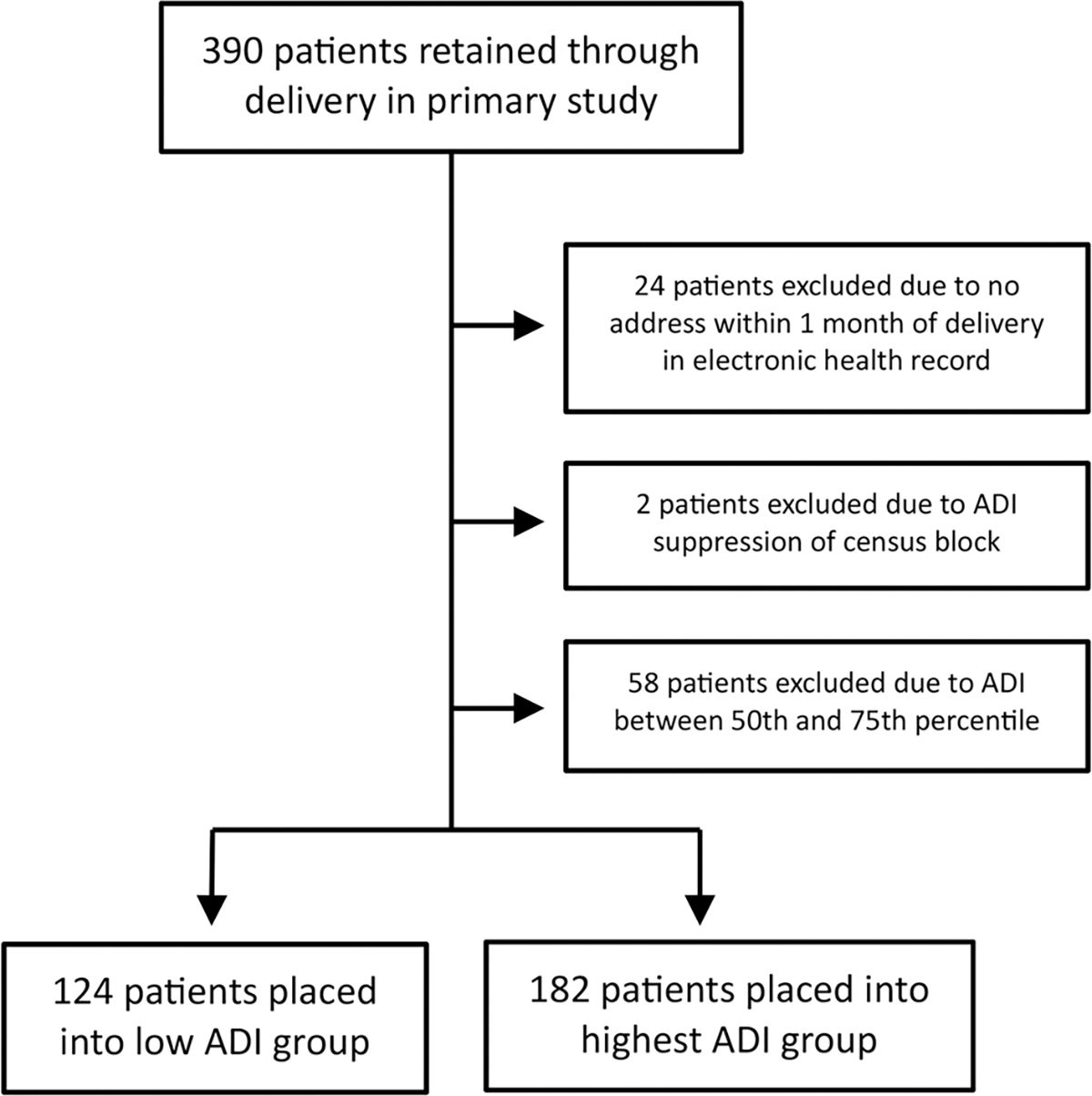
Flow chart of study participants.

Beginning with 390 patients retained through delivery from the primary study, our flow chart of study participants for this secondary study is shown in Figure 1. Of 390 patients, 84 (21.5%) were excluded due to the following reasons: 24 (6.2%) had no address within 30 days of delivery on file in the electronic health record, 2 (0.5%) lived in an area of ADI suppression due to high group quarters population, and 58 (14.9%) had an ADI between the 50th and 75^th^ percentiles. Of the remaining 306 patients, 124 (40.5%) were identified as having an ADI ≤ 50th percentile (median 36, IQR [28,43]), constituting the low ADI group, and 182 (59.5%) were identified as having an ADI ≥ 75th percentile (median 93, IQR [86,97]), constituting the highest ADI group.

There were several differences in baseline characteristics as shown in Table 1. Those with highest ADI (thus highest deprivation) were younger (average age of 27.6 vs 32.6, *p* < 0.01) and more likely to have public insurance (69.2% vs 13.7%, *p* < 0.01). There were significant differences in race distribution, with the highest ADI group being 72.5% Black or African American, while the low ADI group was 75.8% white (*p* < 0.01). There were also significant differences between the groups’ distribution of marital status (*p* < 0.01), highest level of education (*p* < 0.01), and total household income (*p* < 0.01), with the highest ADI group having more patients who are unmarried/unpartnered, have no college degree, and have a total annual household income of less than $15,000. The highest ADI group was less likely to be nulliparous (17.6% vs 37.9%, *p* = 0.02). They also had a higher average BMI (34.5 vs 28.4, *p* = 0.01) and were more likely to be obese (57.1% vs 33.1%, *p* < 0.01) and use tobacco (7.1% vs 1.6%, *p* = 0.03). Of the preexisting medical conditions evaluated, the highest ADI group had higher rates of asthma (16.5% vs 8.9%, *p* < 0.01) and depression (20.9% vs 16.9%, *p* = 0.01). Of the assessed prenatal complications, the only significant difference was higher rates of anemia in the highest ADI group (22.5% vs 14.5%, *p* = 0.04). Notably, the highest ADI group was less likely to have received the COVID-19 vaccination (24% vs 54%, *p* < 0.01).

Rates of perinatal outcomes between groups are shown in Table 2. Although rates of overall preterm deliveries did not differ (23.1% vs 17.7%, *p* = 0.26), the highest ADI group was significantly more likely to deliver before 28 weeks (12.1% vs 4.0%, *p* = 0.01) and before 34 weeks (12.6% vs 5.7%, *p* = 0.04). No significant differences were found in rates of composite or individual maternal complications during delivery, average APGAR scores, or composite neonatal complications following delivery, although the low ADI group had a higher rate of one neonatal complication: intracranial hemorrhage (3.2% vs 0%, *p* = 0.03). There were also no differences in average neonate birth weight.

After adjusting for obesity and tobacco use, lower ADI remained protective against preterm birth for delivery <28 weeks (aOR 0.26, 95% CI 0.07–0.73) and <34 weeks (aOR 0.35 95% CI 0.14–0.87), but not overall (aOR 0.68, 95% CI 0.38–1.244; Table 3). However, when also adjusting for age, ADI was no longer significantly associated with delivery <28 weeks (aOR 0.45 95% CI 0.15–1.37) or <34 weeks (aOR 0.60 95% CI 0.22–1.64).

The highest ADI group was more likely to develop SARS-CoV-2 infection (59.3% vs 41.9%, *p* < 0.01, Table 2) and be hospitalized due to COVID-19 (3.9% vs 0%, *p* = 0.04). The subgroup analysis of perinatal outcomes of those who tested positive for SARS-CoV-2 (52 patients from the low ADI group and 108 patients from the highest ADI group) is shown in Table 4. There were no detectable differences in perinatal outcomes between groups among those with SARS-CoV-2 seropositivity.

Finally, after stratification by race, there were no difference in SARS-CoV-2 infection, average age at delivery, or preterm delivery for Black, White, or Asian individuals in highest or low ADI groups (Table 5).

## Discussion

### Existing research

Race and socioeconomic status are well documented as being associated with differing COVID-19 outcomes and pregnancy outcomes in the United States; Black race and Hispanic ethnicity have been associated with both higher likelihood of SARS-CoV-2 seropositivity and higher severity of COVID-19 in the general population [2,20] and among pregnant patients [21]. Low socioeconomic status has likewise been associated with more severe COVID-19 during pregnancy [22].

Several other neighborhood and sociodemographic characteristics have also been associated with these outcomes. For example, neighborhoods with higher rates of deprivation, as indicated by factors of household income, poverty rates, high school degree attainment, federal financial assistance requirements, proportion of vacant housing, and lack of medical insurance) and higher rates of crowding were found to have higher rates of SARS-CoV-2 seropositivity in pregnant patients [21]. Hispanic pregnant populations have been hypothesized to be at increased risk for SARS-CoV-2 infection due to higher likelihood of living in multigenerational and multi-family homes [23]. Rural communities may demonstrate a stronger correlation between neighborhood deprivation and COVID-19 prevalence than urban communities [14]. Maternity care deserts, especially disparities in access to high-risk obstetric care, as well as food deserts likely worsened during the COVID-19 pandemic, contributing to exacerbation of health inequities [24–26]. Finally, pregnant patients who work in food services and those who have higher rates of occupational exposure to SARS-CoV-2 appear to have more severe infections [22,27].

When looking at ADI specifically, more deprived neighborhoods continue to be associated with worse COVID-19 outcomes and pregnancy outcomes. Across U.S. counties, ADI correlated positively with COVID-19 prevalence [1,14]. In Washington D.C., higher ADI was associated with increased early SARS-CoV-2 transmission rates [15]. At three children’s hospitals across the United States, higher ADI was associated with more severe pediatric COVID-19^16^, reflecting our high rates of COVID-19 hospitalization. Higher ADI has been associated with higher rates of the following: elevated hemoglobin A1C when entering pregnancy [13], post-induction cesarean delivery [7], postpartum readmission [12], development of postpartum Stage 2 hypertension [6], and early breastfeeding cessation in patients with maternal cardiac disease [28]. Newborns born to women living in neighborhoods with higher ADI may be at increased risk for abnormal birth weights [29] and higher morbidity and mortality in Neonatal Intensive Care Units [30].

One prior study has investigated perinatal outcomes during the COVID-19 pandemic using ADI: Yu et al. compared rates of perinatal complications in Michigan before and during the pandemic, finding that Black patients in the most deprived neighborhoods (those with ADI in the highest tertile) had significantly increased rates of severe maternal mortality during the pandemic compared to the three years prior to the pandemic [9]. Their findings support our hypothesis that social and structural barriers to healthcare worsened during the pandemic, widening disparities for our most vulnerable patient populations.

### Interpretation of results

Our primary outcome of overall preterm birth did not differ between low and highest ADI groups; however, high deprivation was significantly associated with higher rates of preterm birth before 34 weeks and extremely preterm birth (<28 weeks). This difference remained even after accounting for cofounders of obesity and tobacco use, two variables also associated with early delivery, but no longer remained if age was also controlled. This correlation is likely due to a combination of social and structural determinants of health associated with high deprivation, as well as higher rates of SARS-CoV-2 infection [31,32]. Among secondary outcomes, intracranial hemorrhage, usually associated with preterm delivery and more common among minority patients [33], was unexpectedly found to be more common among newborns born to women in the low ADI group, although low sample size make conclusions challenging for this outcome.

We found a higher rate of SARS-CoV-2 infection and hospitalization due to COVID-19 in our highest ADI group, which is unsurprising given this group’s lower rates of prior COVID-19 vaccination. Interestingly, among those with SARS-CoV-2 seropositivity, there were no differences in perinatal outcomes between the highest and low ADI groups. This may reflect the delivery hospitals, which are major urban centers where all new interventions, such as the COVID-19 vaccination or monoclonal antibody therapy, were immediately available to all patients, and strict evidence-based care for COVID-19 was followed in treatment with steroids and antibiotics [34].

When stratifying the outcomes of SARS-CoV-2 infection and preterm delivery by race, there were no significant differences found for any race. Rates of preterm delivery in Black patients were 20.5% in the highest ADI group and 9.1% in the low ADI group (*p* = 0.35); conversely, rates of preterm delivery in White patients were 20.0% in the highest ADI group and 21.3% in the low ADI group (*p* = 0.87). Larger sample sizes may allow us to further understand how racism may augment discordant outcomes between those with low and high ADI.

We also found many significant differences in baseline characteristics between the low and highest ADI groups; unsurprisingly, some of these characteristics are variables that are used to calculate ADI. Considerable differences in race between the low and highest ADI groups is also unsurprising due to systemic oppression and marginalization of Black communities in the United States. Lower rates of nulliparity in the highest ADI group may be a result of differences in access to and knowledge of contraception options [35–38]. Obesity [39], asthma [40], anemia [41], and depression [42–44] likewise have been found to disproportionately impact patients of color and those with low socioeconomic status; these disparities reflect contributing factors of poor air and food quality, high levels of stress, and barriers to primary care outside of pregnancy, particularly for patients who are publicly insured such as the majority of our highest ADI group.

Finally, significantly fewer patients in the highest ADI group reported receiving the COVID-19 vaccination, following national trends in vaccine uptake based on race, education level, and income [45]. In a national sample, mistrust due to historic racism and discrimination in medicine impacted patients’ willingness to receive the COVID-19 vaccine [46]. Pregnant patients may be particularly likely to experience vaccine hesitancy, especially regarding novel treatments that may be perceived as understudied or unsafe in pregnancy [47]. Vaccine hesitancy, in addition to disparities in access to vaccination and access to healthcare overall, therefore may contribute to lower vaccination rates in the highest ADI group.

### Clinical implications

Although collection of medical history and comorbidities is routine in obstetric care to best formulate a medical treatment plan, the routine collection of social drivers of health for intervention is unmapped. Overall, our outcomes reflect the necessity of targeting social drivers of health in obstetric care and the need for efficacious interventions that can mitigate their impact and improve health for both the mother and infant.

### Research implications

The Area Deprivation Index is a powerful tool that provides a national scale for access to resources, which is especially useful in investigating multifactorial determinants of health. Tools such as these have potential to help us better understand perinatal care overall and identify targets to mitigate disparate outcomes. In our study, we identified modifiable characteristics for both individuals (such as tobacco use, obesity, and vaccination rates) and communities (partner support, education, and income). Future studies that introduce interventions to modify these targets could thus effectively improve perinatal outcomes such as extremely preterm birth and severe infection for patients with high deprivation.

### Strengths and limitations

This analysis had many strengths, the most notable being a large sample size as well as the use of a prospective cohort during the early COVID-19 pandemic with detailed background, obstetric, and COVID-19 variable collection. This study was also distinct in investigating a wide range of detailed sociodemographic characteristics and both perinatal and COVID-19 outcomes, as well as further evaluating the role that SARS-CoV-2 seropositivity and race may play in impacting these outcomes. Finally, this analysis is one of the first to apply ADI to perinatal outcomes during the early COVID-19 pandemic [9].

Limitations include small sample size for rare outcomes and analysis of preterm birth as a single variable (encompassing both spontaneous and iatrogenic preterm births). Those living in overcrowded areas are excluded from the ADI database [10,11]; these patients may represent an especially disadvantaged group and warrant further study. We did not stratify Sars-CoV-2 seropositivity by gestational age or trimester at the time of infection; further stratification would certainly provide more robust information regarding the effect of Sars-CoV-2 infection on perinatal outcomes, as others have investigated [48]. Furthermore, our study is limited to two urban tertiary care centers in the American Midwest, curbing our generalizability.

## Conclusions

Overall, these findings highlight the complex interplay of factors impacting perinatal outcomes during the COVID-19 pandemic. Clear differences between ADI groups existed in COVID vaccine uptake and therefore likelihood of contracting SARS-CoV-2 infection and being hospitalized for the infection, as well as preterm delivery before 34 and 28 weeks. Future work testing individual and community interventions could mitigate the impact that neighborhood deprivation exerts on outcomes.

## Figures and Tables

**Table 1.** Characteristics of pregnant patients during the COVID-19 pandemic, by ADI.

	ADI ≤ 50th percentile	ADI ≥ 75th percentile	
	(*n* = 124)	(*n* = 182)	*p* value

Average Age (mean ± SD)	32.56 ± 5.3	27.57 ± 5.6	<0.01
Insurance Coverage			
Public	17 (13.7%)	126 (69.2%)	<0.01
Private	104 (83.9%)	49 (26.9%)	<0.01
Uninsured	3 (2.4%)	5 (2.8%)	0.86
Race			<0.01
Black or African American	11 (7.3%)	132 (72.5%)	
Asian	15 (12.1%)	2 (1.1%)	
White	94 (75.8%)	40 (22.0%)	
Other race	4 (3.2%)	8 (4.4%)	
Ethnicity			
Of Hispanic, Latino, or Spanish origin	3 (2.4%)	1 (0.6%)	0.31
Marital Status	9 (7.3%)		<0.01
Single	97 (78.2%)	56 (30.8%)	
Partnered/Married	1 (0.8%)	44 (24.2%)	
Divorced/Separated	0 (0.0%)	4 (2.2%)	
Widowed		0 (0.0%)	
Highest Level of Education Completed			<0.01
Less than high school graduate	3 (2.4%)	18 (9.9%)	
High school graduate or GED	12 (9.7%)	73 (40.1%)	
Bachelor's degree	26 (21.0%)	9 (4.9%)	
Other advanced degree (Master's, Doctoral)	66 (53.2%)	7 (3.8%)	
Total Household income			<0.01
Less than $15,000	3 (2.4%)	37 (20.3%)	
$15,000–24,999	3 (2.4%)	13 (7.1%)	
$25,000–49,999	9 (7.3%)	18 (9.9%)	
$50,000–74,999	16 (12.9%)	9 (4.9%)	
$75,000–99,999	14 (11.3%)	4 (2.2%)	
$100,000 and above	54 (43.4%)	5 (2.7%)	
Nulliparity	47 (37.9%)	32 (17.6%)	0.02
Multiple gestation pregnancy	7 (5.6%)	3 (1.6%)	0.10
Average Body Mass index (mean ± SD)	28.43 ± 6.1	34.5 ± 2.28	0.01
Obesity	41 (33.1%)	104 (57.1%)	<0.01
Current tobacco use	2 (1.6%)	13 (7.1%)	0.03
Preexisting medical condition			
Immunocompromised condition	5 (4.0%)	2 (1.1%)	0.27
Autoimmune disease	8 (6.5%)	4 (2.2%)	0.25
Hypertension	7 (5.6%)	10 (5.5%)	0.50
Type 2 diabetes	5 (4.0%)	8 (4.4%)	0.43
Chronic kidney disease	0 (0.0%)	1 (0.5%)	1.00
Cardiovascular disease	1 (0.8%)	1 (0.5%)	1.00
HIV	0 (0.0%)	1 (0.5%)	1.00
Asthma	11 (8.9%)	30 (16.5%)	<0.01
Other chronic lung disease	1 (0.8%)	1 (0.5%)	1.00
Sickle cell anemia	0 (0.0%)	1 (0.5%)	1.00
Depression	21 (16.9%)	38 (20.9%)	0.01
Other mental health disorder	13 (10.5%)	12 (6.6%)	0.77
Alcohol or substance use disorder	0 (0.0%)	3 (1.6%)	0.25
Other chronic condition	4 (3.2%)	5 (2.7%)	1.00
Received COVID-19 vaccination	67 (54.0%)	44 (24.2%)	<0.01
Pregnancy complications			
Gestational diabetes	9 (7.3%)	12 (6.6%)	0.95
Anemia	18 (14.5%)	41 (22.5%)	0.04
IUGR	4 (3.2%)	12 (6.6%)	0.20
Hyperemesis gravidarum	1 (0.8%)	0 (0.0%)	1.00
Preeclampsia/eclampsia	10 (8.1%)	17 (9.3%)	0.57
Gestational hypertension	15 (12.1%)	21 (11.5%)	0.95
Placental abruption	1 (0.8%)	1 (0.5%)	1.00
Preterm labor	3 (2.4%)	1 (0.5%)	0.31
Bleeding during pregnancy	2 (1.6%)	0 (0.0%)	0.18
Fetal hydrops	0 (0.0%)	1 (0.5%)	1.00
Suspected fetal congenital anomaly	0 (0.0%)	3 (1.6%)	0.27
Placenta previa	2 (1.6%)	1 (0.5%)	0.58

Abbreviations: ADI, Area Deprivation Index; GED, General Educational Development; HIV, Human Immunodeficiency Virus; IUGR, Intrauterine growth restriction.

**Table 2.** Perinatal and COVID-19 outcomes during the COVID-19 pandemic, by ADI.

	ADI ≤ 50th percentile	ADI ≥ 75th percentile	
	(*n* = 124)	(*n* = 182)	*p* value

Average gestational age at delivery (mean (IQR))	39 (37,39)	38 (37,39)	0.06
Preterm delivery (all)	22 (17.7%)	42 (23.1%)	0.26
Before 28 weeks	5 (4.0%)	22 (12.1%)	0.01
Before 34 weeks	7 (5.7%)	23 (12.6)	0.04
Mode of delivery			0.11
Vaginal delivery	78 (62.9%)	112 (61.5%)	
Operative vaginal delivery	2 (1.6%)	7 (3.9%)	
Scheduled Cesarean section	16 (12.9%)	25 (13.7%)	
Unscheduled Cesarean section	24 (19.4%)	17 (9.3%)	
Maternal complications during delivery (composite)	19 (15.3%)	16 (8.8%)	0.08
Hemorrhage	13 (10.5%)	11 (6.0%)	0.21
Chorioamnionitis or endomyometritis	2 (1.6%)	1 (0.5%)	0.58
Shoulder dystocia	3 (2.4%)	4 (2.2%)	1.00
Blood transfusion	2 (1.6%)	1 (0.5%)	0.58
Maternal death	0 (0.0%)	0 (0.0%)	–
Average APGAR scores (mean (IQR))			
1 minute	8 (7,8)	8 (8,8)	0.70
5 minutes	9 (9,9)	9 (9,9)	0.12
Average neonate birth weight (mean ± SD)	3168.2 ± 655.8	3086.9 ± 604.1	0.28
<2500g	18 (14.5%)	39 (21.4%)	0.13
<1500g	7 (5.7%)	22 (12.1%)	0.06
Neonatal complications following delivery (composite)	14 (11.3%)	17 (9.3%)	0.58
Necrotizing enterocolitis	1 (0.8%)	1 (0.5%)	1.00
Chromosomal abnormality	3 (2.4%)	0 (0.0%)	0.07
Intracranial hemorrhage	4 (3.2%)	0 (0.0%)	0.03
NICU admission	13 (10.5%)	16 (8.8%)	0.80
Neonatal death	0 (0.0%)	2 (1.1%)	0.51
SARS-CoV-2 infection during pregnancy	52 (41.9%)	108 (59.3%)	<0.01
Hospitalized due to COVID-19	0 (0.0%)	7 (3.9%)	0.04
Number of days hospitalized (mean (IQR))	0 (0,0)	3 (1,4)	–

Abbreviations: ADI, Area Deprivation Index; APGAR, Appearance, Pulse, Grimace, Activity, and Respiration; NICU, Neonatal Intensive Care Unit.

**Table 3.** Multivariable regression of primary outcome.

	ADI <50% aOR (95% CIs)

Preterm birth (with variables age, obesity, tobacco use)	0.62 (0.31, 1.21)
Before 28 weeks	0.45 (0.15, 1.37)
Before 34 weeks	0.60 (0.22, 1.64)
Preterm birth (with variables obesity, tobacco use)	0.68 (0.38, 1.24)
Before 28 weeks	0.26 (0.08, 0.73)
Before 34 weeks	0.35 (0.14, 0.87)

Abbreviations: ADI, Area Deprivation Index; aOR, adjusted odds ratio; CI, confidence interval.

**Table 4.** Subgroup analysis of SARS-CoV-2-positive subgroup.

	ADI ≤ 50th percentile	ADI ≥ 75th percentile	
	(*n* = 52)	(*n* = 108)	*p* value

Preterm delivery (all)	8 (15.4%)	20 (18.5%)	0.63
Before 28 weeks	1 (1.9%)	6 (5.6%)	0.43
Before 34 weeks	2 (3.9%)	6 (5.6%)	1.00
Mode of delivery			0.39
Vaginal delivery	37 (72.6%)	71 (69.6%)	
Operative vaginal delivery	0 (0.0%)	3 (2.9%)	
Scheduled Cesarean section	6 (11.8%)	18 (17.7%)	
Unscheduled Cesarean section	8 (15.7%)	10 (9.8%)	
Maternal complications during delivery (composite)	9 (17.3%)	13 (12.0%)	0.36
Hemorrhage	7 (13.7%)	8 (7.8%)	0.24
Chorioamnionitis or endomyometritis	1 (2.0%)	1 (1.0%)	1.00
Shoulder dystocia	1 (2.0%)	4 (3.9%)	1.00
Blood transfusion	2 (3.9%)	0 (0.0%)	0.11
Maternal death	0 (0.0%)	0 (0.0%)	--
Average APGAR scores (mean (IQR))			
1 minute	8 (7,8)	8 (8,8)	0.43
5 minutes	9 (8,9)	9 (9,9)	0.11
Average neonate birth weight (mean ± SD)	3192.5 ± 613.1	3097.5 ± 614.8	0.37
<2500 g	7 (13.5%)	20 (18.5%)	0.42
<1500 g	2 (3.9%)	6 (5.6%)	1.00
Neonatal complications following delivery (composite)	7 (13.5%)	10 (9.3%)	0.42
Chromosomal abnormality	2 (3.9%)	0 (0.0%)	0.11
NICU admission	6 (11.8%)	9 (8.7%)	0.55
Neonatal death	0 (0.0%)	1 (1.0%)	1.00

Abbreviations: ADI, Area Deprivation Index; APGAR, Appearance, Pulse, Grimace, Activity, and Respiration; NICU, Neonatal Intensive Care unit.

**Table 5.** Outcomes by race (all patients).

	ADI ≤ 50th percentile	ADI ≥ 75th percentile	
	(*n* = 124)	(*n* = 182)	*p* value

COVID-19 infection during pregnancy, all races	52 (41.9%)	108 (59.3%)	<0.01
Black	5 (45.5%)*	79 (59.9%)*	0.35
White	42 (44.7%)*	22 (55.0%)*	0.27
Asian	4 (26.7%)*	1 (50.0%)*	0.51
Average gestational age at delivery, all races (mean (IQR))	39 (37,39)	38 (37,39)	0.06
Black	39 (37,39)	38 (37,39)	0.59
White	39 (37,39)	39 (37,39)	0.86
Asian	39 (39,39)	37 (37,37)	0.12
Preterm delivery, all races	22 (17.7%)	42 (23.1%)	0.26
Black	1 (9.1%)*	27 (20.5%)*	0.69
White	20 (21.3%)*	8 (20.0%)*	0.87
Asian	0 (0.0%)*	1 (50.0%)*	0.12

Abbreviations: ADI, Area Deprivation Index

* Percentages indicate the percentage of patients experiencing the indicated outcome out of all patients within the ADI race subgroup.

## Data Availability

The data that support the findings of this study are available from the corresponding author, A.B., upon reasonable request.
